# Smartphone-Based Hearing Aid Compression and Noise Reduction

**DOI:** 10.3390/s22093306

**Published:** 2022-04-26

**Authors:** Aoxin Ni, Nasser Kehtarnavaz

**Affiliations:** Department of Electrical and Computer Engineering, The University of Texas at Dallas, Richardson, TX 75080-3021, USA; kehtar@utdallas.edu

**Keywords:** smartphone-based hearing aid compression and noise reduction, compression and noise reduction in the field, real-time platform to study hearing aid functions

## Abstract

This paper presents an assistive hearing smartphone app mimicking the two main functions of hearing aids, consisting of compression and noise reduction. The app is designed to run in real time on smartphones or tablets. Appropriate levels of amplification or gain are activated by selecting a filter from a filter bank for six audio environment situations covering three sound pressure levels of speech and two sound pressure levels of noise. The results of this smartphone app for real-world audio environments are provided, indicating its effectiveness as a real-time platform for studying compression and noise reduction algorithms in the field or in realistic audio environments.

## 1. Introduction

According to the World Health Organization (WHO), the number of people with hearing loss will grow to nearly 2.5 billion people by 2050, with 700 million of them suffering from severe hearing loss [1]. To cope with hearing loss, hearing aids are prescribed by audiologists.

Considering the widespread utilization of smartphones all around the world, hearing aid manufacturers have introduced smartphone apps such as Signia, Starkey Thrive Hearing Control, and Oticon ON [2] for adjustment of some of the parameters of hearing aids for those devices that have Bluetooth wireless connectivity capability. However, hearing aid manufacturers do not allow the signal processing software running on their hearing aid processors to be accessed or modified by researchers for conducting hearing enhancement studies in the field. In this work, we implemented the two key functions of hearing aids—consisting of compression and noise reduction—on smartphones in an open-source manner, so that researchers and audiologists would be able to experiment with different compression and noise reduction algorithms in the field or in real-world audio environments.

**Figure 1:**
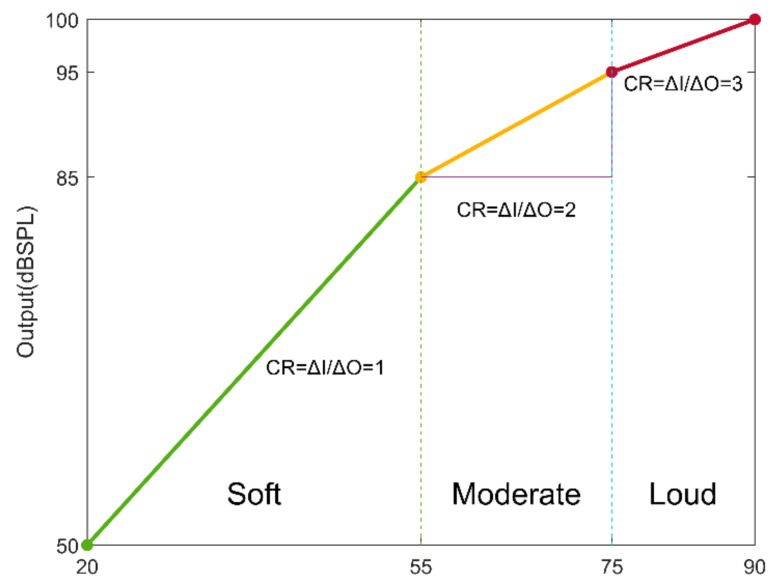
Sample compression curve or compression ratios for different speech intensity levels in a typical frequency band.

Humans have a roughly 120 dB dynamic range of hearing. In people with hearing loss, this range is narrowed or reduced, and it is thus necessary to map the above wide range of sound into a narrower range. This process is referred to as wide dynamic range compression (WDRC). WDRC provides more gain for low-SPL (sound pressure level) signals and less gain for high-SPL signals. The compression can be represented graphically by an input/output compression function or curve for each frequency band. Figure 1 illustrates a typical compression curve, showing how input sound levels are mapped into output sound levels of a hearing aid. Since hearing perception changes depending on the frequency of sounds, different compression curves are applied in different frequency bands [3,4]. Based on different speech SPLs, different compression ratios are applied to soft, moderate, and loud speech sound levels. Similar to [5,6], Wiener filtering noise reduction (NR) is used here together with compression in hearing aids in order to improve hearing in noisy environments. The noise reduction function reduces the background noise level so that the speech sound can be heard more clearly.

Speech sounds with SPL values of less than 55 dB are regarded as soft speech. Speech sounds with SPL values between 55 dB and 75 dB are regarded as moderate speech, while speech sounds with SPL values greater than 75 dB are regarded as loud speech [7]. Depending on these three speech SPL levels, WDRC applies different compression ratios to sound signals in a number of frequency bands. Different numbers of frequency bands are considered. In a typical conventional compression prescription such as the one in [7], nine frequency bands are used. In our experimentations, the NR threshold of 55 dB was found to be the most effective for activating noise reduction. The app allows users to modify the NR threshold in its settings. To take into consideration different speech SPLs as well as different noise SPLs in real-world audio environments, an FIR filter bank consisting of several FIR filters was designed to achieve WDRC and NR at the same time across a number of frequency bands.

For operation in real-world audio environments, a voice activity detector (VAD) module is needed [8,9]. In the smartphone app developed in this work, the VAD described in [10] is used to separate speech frames from noise frames, and an SPL meter described in [11] is used to measure SPL of sound frames. With the VAD and the SPL meter, the app selects an appropriate filter and processes the input audio signal in real time to achieve WDRC and NR. Further details of the developed smartphone app are described in the remaining sections of the paper. Section 2 describes an overview of the WDRC and NR algorithms and the signal processing flowchart of the developed smartphone app. The experimental setup and field testing results are then presented in Section 3, followed by the conclusion in Section 4.

## 2. App Signal Processing Flowchart

**Figure 2:**
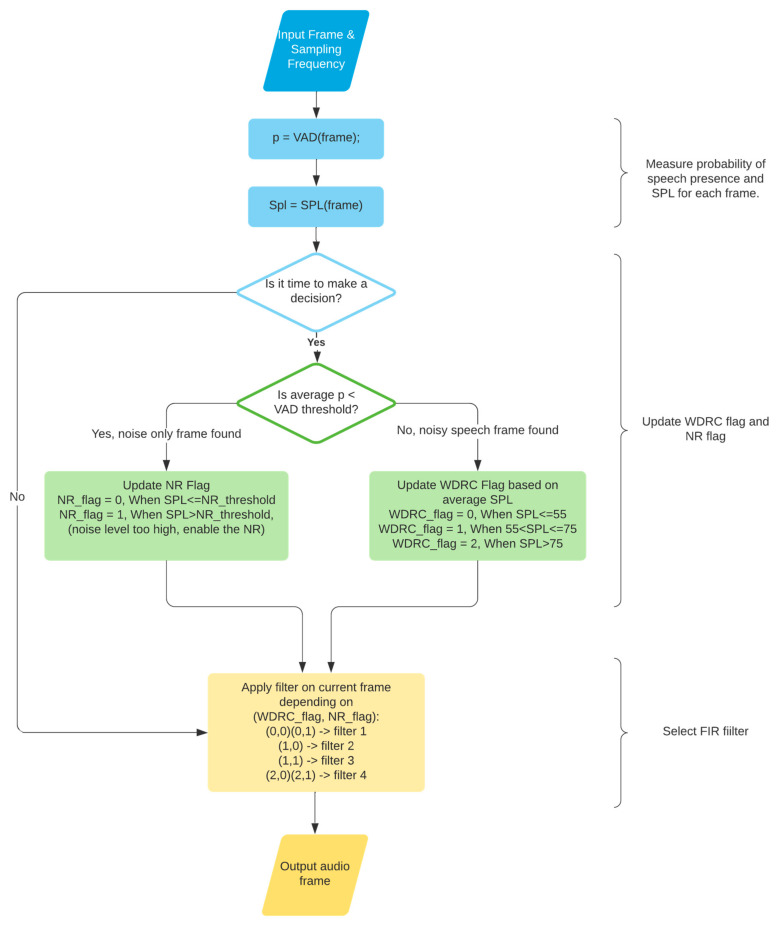
App signal processing flowchart.

Figure 2 depicts the signal processing flowchart of the app, which consists of three parts: VAD and SPL measurement, filter selection, and sound signal filtering. In the first part, the app measures a confidence or probability value for the VAD together with the SPL value of each input sound frame. In the second (filter selection) part, it decides which filter in the filter bank to use at a specified decision rate. The default decision rate is set to 200 frames, which means that a decision is made once every 200 frames. The app allows this rate to be adjusted by the user. The information regarding whether an incoming speech level is soft, moderate, or loud, and whether the noise reduction module should be enabled, is stored in a buffer for making the next filter selection. In the last part, the selected filter is used to process sound signals for the next filter selection time duration, or the next 200 frames. The sampling frequency is set to 48 kHz, since this sampling rate generates the lowest latency on smartphones [12].

The VAD module used in the app is based on the VAD approach described in [10,13], providing the probability of the presence of speech in a frame. By adjusting a threshold in the VAD, the noise-only frames and noisy speech frames are separated according to the probability of the presence of speech in those frames. For SPL measurement, the splmeter function of MATLAB is used to return an average time-weighted sound level in dB [11]. The VAD function and splmeter function are converted to C codes from MATLAB codes by using the Coder utility of MATLAB. The steps to convert MATLAB codes to C codes are covered in [14,15]. The generated C codes are then placed inside a shell, which was previously developed and described in [14]. Interested readers are referred to [16,17] for more details related to embedding MATLAB-generated C codes into the software development environments of smartphones.

In the filter selection part, the VAD value is used to separate noise-only frames from noisy speech frames. When a frame is pure noise and does not contain any speech, a variable called ‘NR_flag’ is updated for the purpose of deciding whether to enable the noise reduction module or not. When a frame contains speech with noise or no noise, a variable called ‘WDRC_flag’ is updated to see whether the speech SPL is soft, moderate, or loud. Then, based on ‘NR_flag’ and ‘WDRC_flag’, a so-called temporary filter selection is made for each frame. All temporary filter selections are saved in a buffer depending on the decision rate. The filter selection is performed by taking majority voting within the buffer.

Six filters were designed for the three settings of WDRC_flag and two settings of NR_flag, as indicated in Table 1. Based on an audiogram, the compression amount is determined. An audiogram indicates the lowest level of sound that is audible to a person in a number of frequency bands. A sample audiogram used in this paper for WDRC implementation is shown in Table 2.

**Figure 3:**
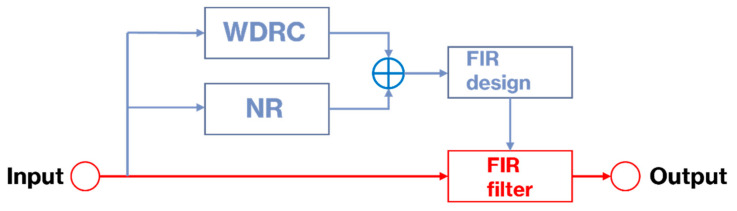
Filter design and filter operation paths.

The WDRC or compression gain values are then generated based on a prescriptive compression fitting [18,19]. In our implementation, the nine bands that are commonly used for compression are mapped into five bands in order to make the computation time more efficient and, thus, achieve real-time processing. Performance-wise, we found that there was not much difference when using five bands as compared to nine bands. Table 3 shows the compression ratio and makeup gains derived based on [19] by using the audiogram in Table 2 as the input. The nominal values of the other compression parameters for performing the WDRC are shown in Table 4. The NR algorithm used in the app is the one described in [20]. Here, it is worth pointing out that the app was designed in a modular manner, which means that the above compression and noise reduction algorithms in the app can be easily replaced by any other compression and noise reduction algorithms. One way this can be achieved is by designing a new filter bank corresponding to these algorithms. Then, our filter bank part of the app can be replaced by the new filter bank within the jni (Java Native Interface) folder of the app. Another way involves substituting other compression and noise reduction algorithms written in C within the jni folder of the app by following the steps mentioned in [14]. As illustrated in Figure 3, the WDRC and NR modules are placed in parallel. The six filters or the filter bank are designed according to the six settings of the WDRC and NR flags.

**Figure 4:**
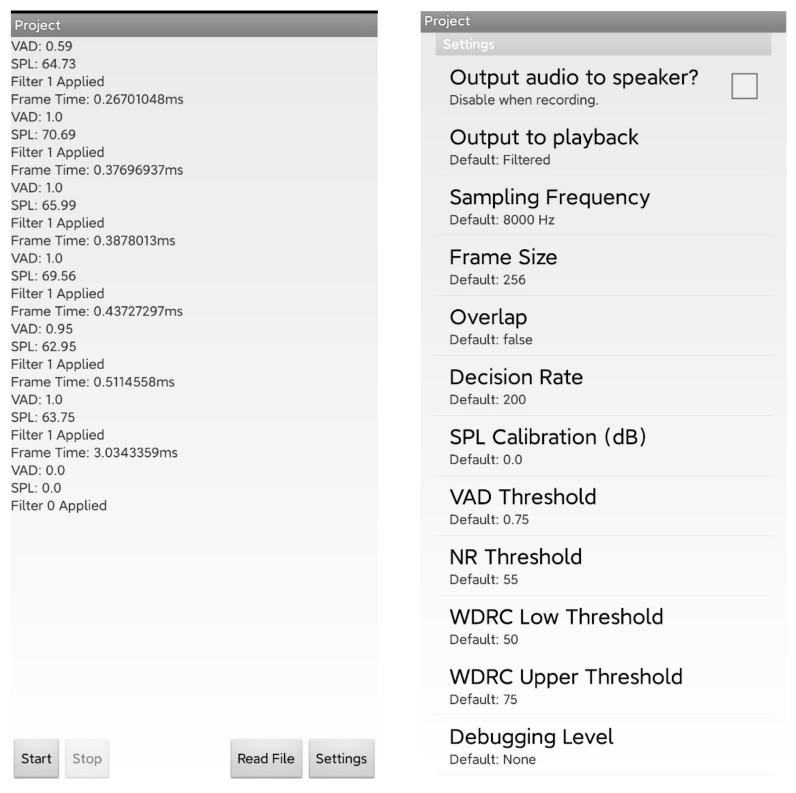
The app’s graphical user interfaces.

The app has two main graphical user interfaces: one is the operation interface, and the other is the settings interface (see Figure 4). In the operation interface, the start button runs the app in real time, while the “read file” button runs the app by processing a stored audio file. In the settings interface, the user can control how the output plays, change the sampling frequency, and set a debugging option. For a more accurate SPL value, users can specify a calibration value for the device in the app’s settings by comparing and matching the computed SPL value with the one indicated by an SPL meter. When the app runs in real time or on a stored audio file, the VAD value, SPL value, and selected filter number for each frame are displayed in a rolling-up manner. The debugging option can be set to store the above information.

## 3. Results of Real-World Audio Environment Experiments

The code for the entire signal processing pipeline is written in C within a project shell described in [14], based on the Android Studio SDK (Software Development Kit) and Android NDK (Native Development Kit) [21]. It is worth noting that there exists a similar shell for iOS, which is also described in [14]. The app code is available for public use at the website stated in [22].

Experiments were conducted by running the app with a frame size of 256 samples at a sampling frequency of 48 kHz on an Android phone with a Snapdragon processor. To obtain the quantitative results reported in this section, audio files of real-world noisy audio environments were captured and stored on the phone’s storage. By running the app on the audio files, the filtered audio and a text file were generated, which contained the VAD, SPL, and selected filter values in the same folder where the audio file was stored. The filtered audio and text files were then used to report the results discussed next.

**Figure 5:**
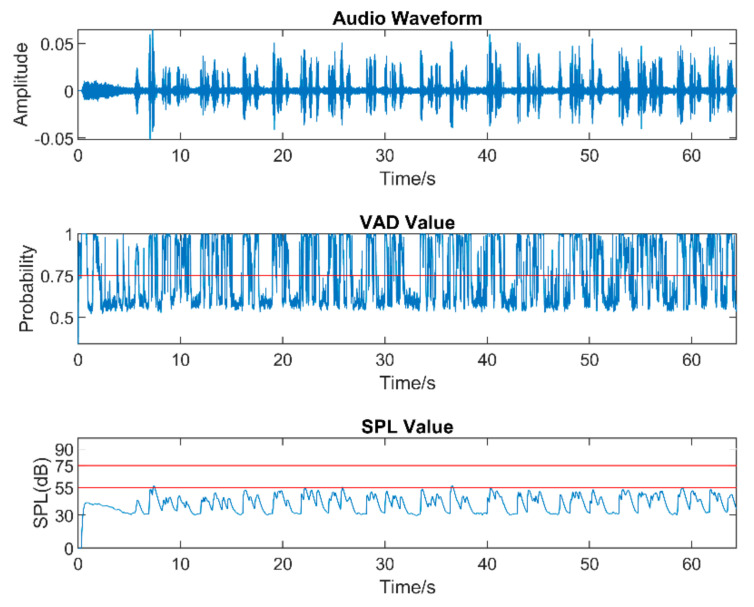
Sample of a soft−speech audio.

**Figure 6:**
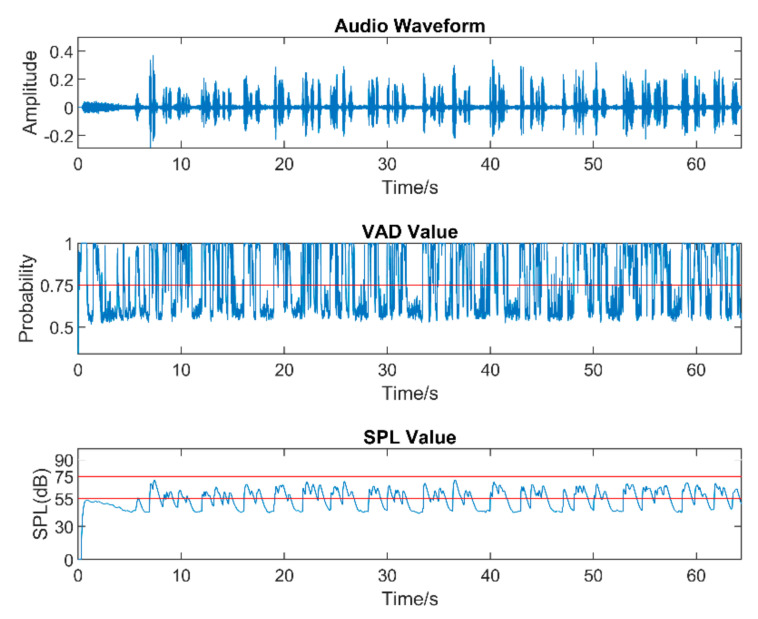
Sample of a moderate−speech audio.

**Figure 7:**
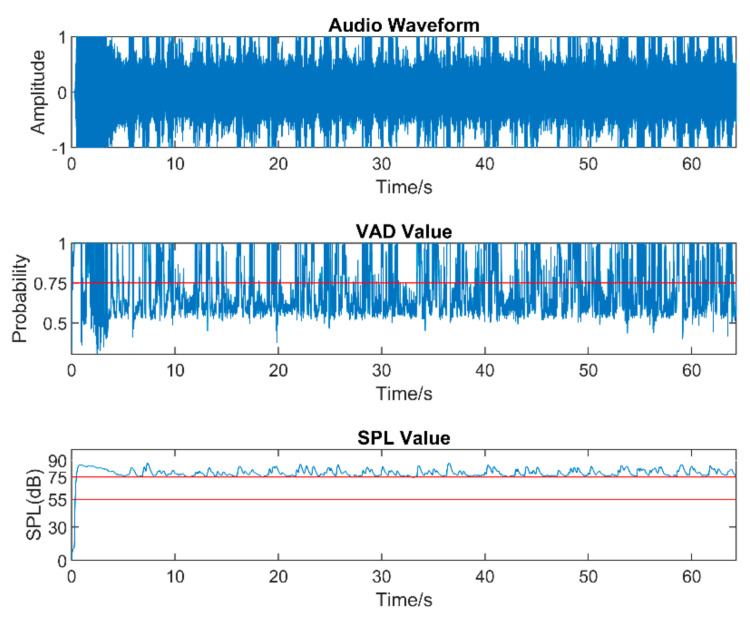
Sample of a loud−speech audio.

Real-world audio files were captured at a 48 kHz sampling frequency in the presence of commonly encountered noisy environments, such as dining halls, lecture rooms, machinery, cars, streets, etc. Table 5 lists the number of audio files collected in different audio scenarios. During audio sound capture, the environmental noise was recorded first for a few seconds. The purpose of doing so was for the app to detect the initial noise conditions for the speech to follow. The signal-to-noise ratio (SNR) was altered by changing the distance between the phone and the person talking. In other words, this allowed different levels of speech to be captured in the noisy environments. As a result, different filters needed to be used across a single audio file. The input audio signal is normalized in the app before processing to make sure that the same output values are generated when using different smartphones. Figure 5, Figure 6 and Figure 7 exhibit the waveforms, VAD values, and SPL values of three sample audio files. These three audio files were recorded in the presence of an air purifier, which is a fan noise type. Based on the VAD values, the speech parts were separated from the noise-only parts. Furthermore, as per the SPL values, the speech level was taken to be soft, moderate, or loud. The app applied different filters from the filter bank depending on the VAD and SPL values.

To verify that the filter bank could perform the compression (WDRC) and the noise reduction (NR) properly, an experiment was conducted by considering only a single filter and by speaking the numbers 1 through 10 in a counting order with washing machine noise in the background. This allowed an easy separation between speech and background noise. As expected, the SPL of the audio signal increased because of the gain applied by the WDRC, and the noise level was decreased when the NR was activated.

**Figure 8:**
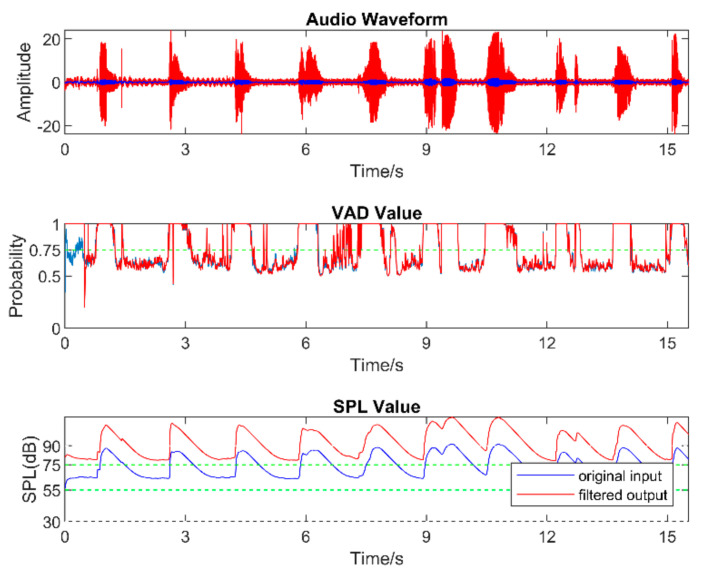
Outcome when Filter 1 was applied.

**Figure 9:**
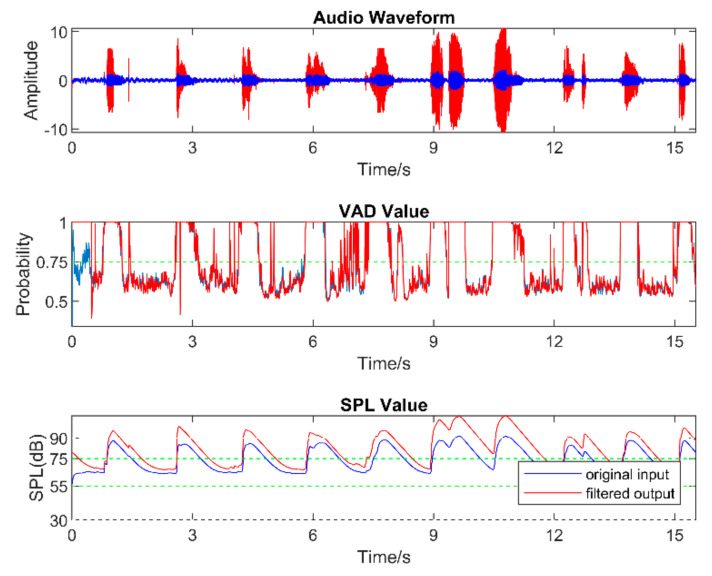
Outcome when Filter 2 was applied.

**Figure 10:**
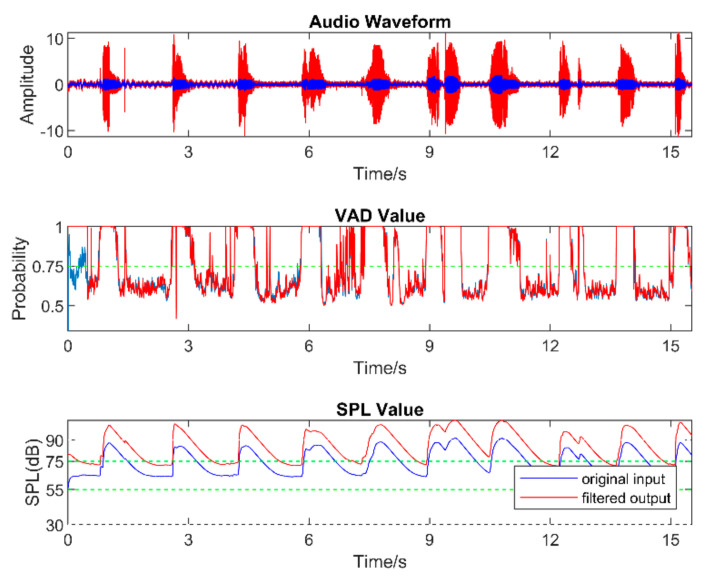
Outcome when Filter 3 was applied.

**Figure 11:**
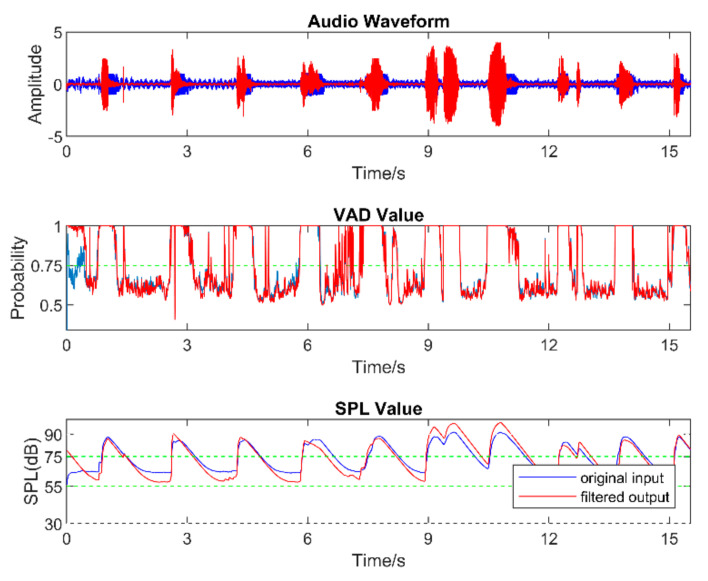
Outcome when Filter 4 was applied.

**Figure 12:**
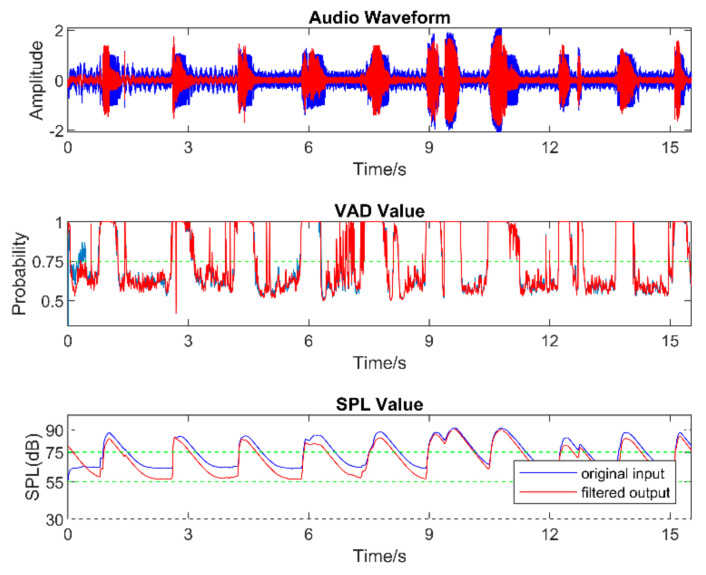
Outcome when Filter 5 was applied.

**Figure 13:**
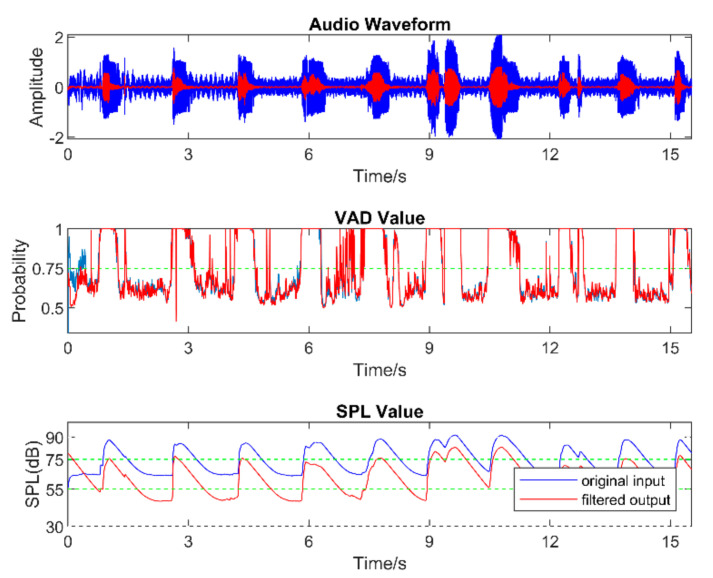
Outcome when Filter 6 was applied.

Figure 8, Figure 9, Figure 10, Figure 11, Figure 12 and Figure 13 illustrate the effect of applying the six filters in the filter bank. In these figures, the blue lines correspond to the original audio signals, while the red lines correspond to the filtered audio signals. By comparing the effects of Filter 1, Filter 3, and Filter 5, which were designed for soft, moderate, and loud speech levels, respectively, it can be seen that the app applied a relatively larger gain on the audio signal when Filter 1 was selected, a smaller gain when Filter 3 was selected, and the smallest or even negative gain when Filter 5 was selected.

Furthermore, by comparing the effects of Filters 1 and 2, Filters 3 and 4, and Filters 5 and 6, which corresponded to situations with the noise reduction on and off, it can be seen that the SPL of the noise-only part was suppressed when the noise reduction was enabled. For the soft-speech audio, even though the speech was amplified by the WDRC, the SPL of the noise part was kept around the original level. For the loud-speech audio, due to the very small gain applied by the WDRC, the SPLs for both the speech and the noise were reduced considerably, which naturally impacted the quality of the speech signal. In all of the experiments carried out in various realistic audio environments, it was found that the compression and noise reduction were carried out according to the designed filters for those audio environments.

**Figure 14:**
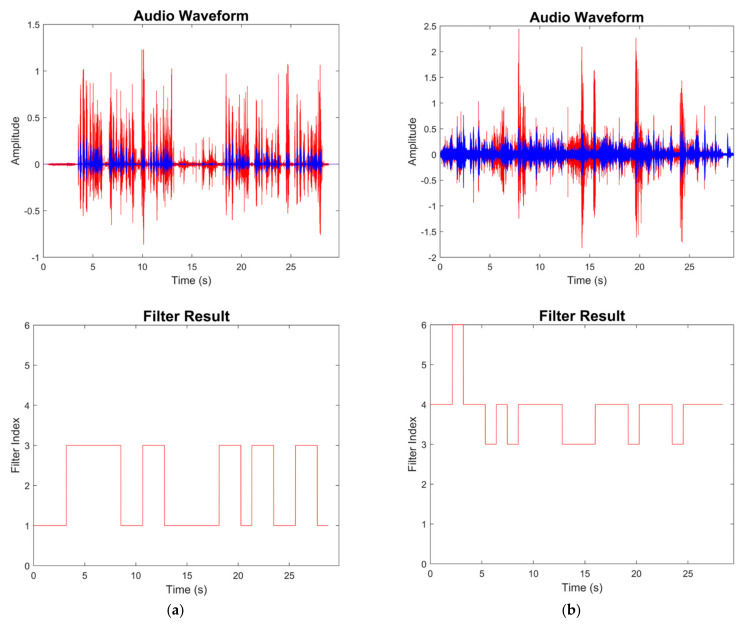
Sample audio signal: (**a**) study room; (**b**) driving.

**Figure 15:**
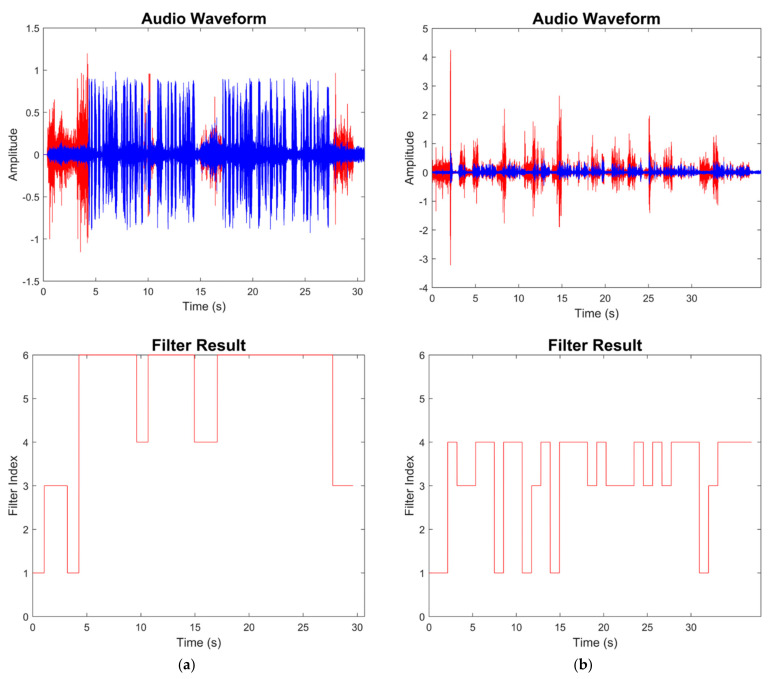
Sample audio signal: (**a**) open air; (**b**) dryer turning on.

Figure 14 and Figure 15 show the results for four sample audio signals illustrating the difference between the original audio and filtered audio waveforms. The filter result graph outcome in Figure 14a indicates that the SPL of the speech in this audio signal was at the soft-to-moderate level. As a result, the WDRC applied a large gain to this audio signal. Figure 14b indicates that Filter 3 and Filter 4 were applied for most of the audio signal, which meant this audio signal contained a moderate speech level with both high and a low noise levels. It can be seen that the waveform peak was higher when Filter 3 was selected, and Filter 4 was selected to achieve noise reduction. The effect of noise reduction can also be observed in Figure 15a,b, which show that the noise reduction was activated for most of the audio signal.

**Figure 16:**
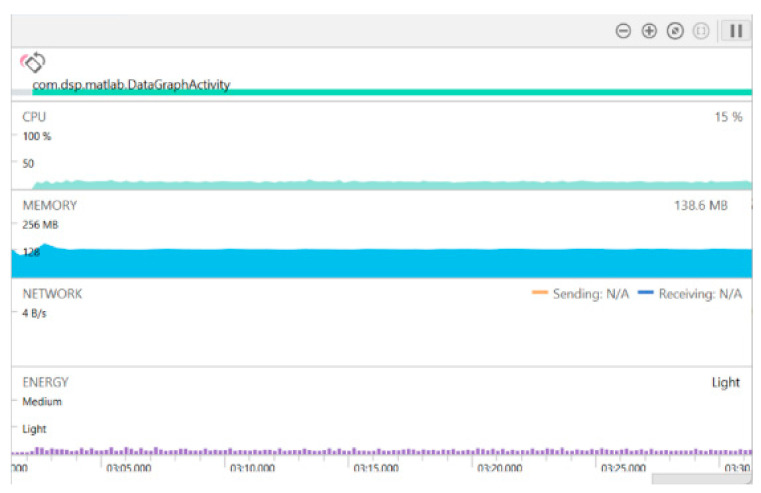
Developed app memory and CPU usage.

For typical hearing improvement smartphone apps, the latency needs to remain below 20 ms [23,24] in order not cause a mismatch between hearing the voice(s) and seeing lip movements. At a sampling frequency of 48 kHz and a frame length of 256 samples, the maximum allowable time is about 5 ms. A real-time throughput is thus achieved, and no downsampling is needed, since the processing time per frame is about 1.05 ms, which is well below 5 ms. In the app, the audio processing buffer is set to be twice the input frame size, so as to enable a 50% overlap between consecutive signal frames. Figure 16 shows the memory and CPU usage of the app on the Android smartphone used.

## 4. Conclusions

In this paper, a real-time smartphone app was developed to mimic the two key functions of hearing aids, consisting of compression and noise reduction. The developed app allows a smartphone to be used as a virtual hearing aid for the purpose of studying the compression and noise reduction functions of hearing aids in the field, or in realistic audio environments, noting that hearing aid manufacturers do not allow access to these signal processing modules on their hearing aids. The open-source nature of this app allows researchers and audiologists to study and experiment with different compression and noise reduction algorithms.

## Figures and Tables

**Table 1:** Filter index for WDRC and NR flag settings.

Filters	Filter 1	Filter 2	Filter 3	Filter 4	Filter 5	Filter 6
WDRC	Soft	Soft	Moderate	Moderate	Loud	Loud
NR	Off	On	Off	On	Off	On

**Table 2:** Sample audiogram for WDRC implementation.

Frequency Bands (Hz)	125–250	250–500	500–750	750–1000	1000–1500	1500–2000	2000–3000	3000–4000	4000–6000
Audiogram (dB SPL)	20	25	30	30	35	35	40	35	35

**Table 3:** Compression ratios and makeup gains for the WDRC implementation.

Speech Intensity	Compression Ratio	Makeup Gains (dB)
Soft Speech	[1.0 1.0 1.0 1.0 1.0]	[10.0, 11.0, 14.0, 25.0, 21.0]
Moderate Speech	[1.6 2.0 1.6 1.6 5.0]	[8.0, 9.0, 13.0, 23.0, 19.0]
Loud Speech	[3.0 2.8 5.0 20.0 20.0]	[6.0, 4.0, 8.0, 18.0, 15.0]

**Table 4:** Nominal values of the other compression parameters for the WDRC implementation.

Release Time	Attack Time	Compression Threshold	Knee Width
1 s	0.01 s	−45 dB	5 dB

**Table 5:** Number of audio files collected from real-world audio environments (this audio dataset is available for public use at the website stated in [22]).

Noise Type	Audio Environment	Number of Audio Files
Machinery Noise	Dryer, shaver, washing machine	13
Wind Noise	Open air, parking lot	12
Car Noise	Bus, car driving	15
Babble	Cafeteria, party, dining hall, campus	21
Music	Song, piano, radio	4
Water Flow	Sink, raining	4
No Noise or Quiet	Study room	14

## Data Availability

The audio dataset used in this paper can be found by following Github link: https://github.com/SIP-Lab/Smartphone-HearingAid-Compression-NoiseReduction (accessed on 15 February 2022).
